# The Role of Highly Active Antiretroviral Therapy (HAART) on Interleukin 17A (IL-17A) in Normotensive and Preeclamptic Black South African Women

**DOI:** 10.1155/2020/3417632

**Published:** 2020-05-30

**Authors:** Wendy N. Phoswa, Thajasvarie Naicker, Veron Ramsuran, Jagidesa Moodley

**Affiliations:** ^1^Discipline of Obstetrics and Gynecology, Nelson R. Mandela School of Medicine, University of KwaZulu-Natal, Durban, South Africa; ^2^Optics and Imaging Centre, University of KwaZulu-Natal, Durban, South Africa; ^3^KwaZulu-Natal Research Innovation and Sequencing Platform (KRISP), School of Laboratory Medicine and Medical Sciences, University of KwaZulu-Natal, Durban, South Africa; ^4^Centre for the AIDS Programme of Research in South Africa (CAPRISA), Durban, South Africa; ^5^Women's Health and HIV Research Group, University of KwaZulu-Natal, Durban, South Africa

## Abstract

**Introduction:**

Interleukin 17A has been implicated in the pathophysiology of both human immune deficiency virus and preeclampsia. This study evaluated serum levels of IL-17A based on pregnancy type, gestational age, HIV status, and duration of HAART. *Material and Methods*. A sample size of 250 was analysed: normotensives (*n* = 150; N) and preeclamptics (*n* = 100; PE). Normotensives were further stratified into HIV negative (*n* = 90), HAART-acute (*n* = 30), and HAART-chronic (*n* = 30). The PE group was divided into early onset (*n* = 50; EOPE) and late onset (*n* = 50; LOPE). The EOPE and LOPE groups were subdivided into HIV negative (*n* = 30), HAART-acute (*n* = 10), and HAART-chronic (*n* = 10). Analysis of IL-17A was performed using a multiple Bio-Plex immunoassay method.

**Results:**

Pregnancy type: the levels of IL-17A were increased in PE compared to N (*P* = 0.0014). Gestational age: the levels of IL-17A were increased in EOPE compared to N group (*P* = 0.0113). A significant increase in the levels of IL-17A in LOPE compared to N was observed (*P* = 0.0063). HIV status: the levels of IL-17A were increased in PE compared to N (*P* = 0.0114) and in EOPE compared to N groups (*P* = 0.0071). HAART duration: the concentration of IL-17A was increased in HAART-chronic PE compared to N groups (*P* = 0.0062). There was also an increase in the levels of IL-17A in EOPE compared to N (*P* = 0.0029).

**Conclusion:**

The study demonstrates that IL-17A is involved in the pathophysiology of PE and that in the presence of HIV infection, chronic HAART administration predisposes women to the development of EOPE.

## 1. Introduction

Preeclampsia (PE) is a pregnancy-specific, multisystemic disorder that occurs in 2-8% of all pregnancies worldwide [1]. Preeclampsia usually presents with the clinical signs of new-onset hypertension after 20 weeks of gestation [2, 3]. Currently, there is no cure for PE with the only effective resolution being delivery, and even then, PE may continue in the postpartum period or present de novo. Furthermore, women with PE as well as their children are at a greater risk of developing chronic diseases including cardiovascular diseases in later life [4]. Nonetheless, much progress has been made in the understanding of the pathophysiology of PE; abnormal placental development characterized by inadequate spiral artery remodelling maybe the primary factor that leads to pathogenesis of the disease. This lack of physiological conversion of the spiral artery leads to a decreased uteroplacental blood flow. In response to hypoxia, the placenta releases various substances including trophoblastic debris and apoptotic cells. These substances cause an imbalance between angiogenic and antiangiogenic factors which contribute to widespread endotheliosis leading to vasospasm, hypertension, and multiple organ affectation [2, 4].

Environmental, genetic, and immunological factors may initiate/exacerbate the cascade of events that lead to placental dysfunction. In addition, the frequency of PE may be affected by HIV infection. Both PE and HIV infection are currently the leading causes of maternal and fetal deaths in sub-Saharan countries [5, 6].

The current recommended treatment for HIV infection is highly active antiretroviral therapy (HAART) (4). However, HAART may alter the immune response of HIV-infected pregnant women thereby predisposing them to the development of PE (4). Nonetheless, inflammatory cytokines (pro- and anti-inflammatory) play a vital role in the pathophysiology of both conditions.

During the pathophysiology of PE, there is a shift from anti-inflammatory (Th2) to a proinflammatory response (Th1) with an elevation of IL-2, IL-6, IL-12, IFN-*γ*, and TNF-*α*. Several studies have found increased levels of these cytokines in PE [7–11]. During HIV infection, there is a shift from Th1 to Th2 immune response. However, this shift is counteracted by HAART [12]. Studies have documented that HIV-infected women have a higher risk of the development of PE compared to HIV-naïve women, postulated to emanate from the immune reconstitution effects of HAART [13–15].

Since both PE and HIV are immune/inflammatory conditions, exaggerative activation or reduction of Th1/Th2 cytokines has been reported by several studies to be strongly associated with the pathophysiology of these disorders [7, 16–18]. Amongst these cytokines, the role of IL-17 in the pathophysiology of both conditions still remains controversial; no studies have been reported on the role of IL-17A in HIV-infected women who develop PE.

Interleukin IL-17A is a proinflammatory cytokine involved in the pathophysiology of both PE and HIV infection. Increased concentrations of IL-17A have been shown to be responsible for the activation of inflammatory responses in PE [19, 20]. In addition, IL-17A has been associated with the development of PE through induction of proinflammatory cytokines such as TNF-*α*, IL-1*β*, IL-6, and IL-8 [21]. In contrast, a study by Wu et al. suggested a beneficial function for IL-17A during pregnancy including the promotion of embryo implantation and maintenance of pregnancy [22]. Additionally, during the progression of HIV infection, IL-17A declines in both human and animal models [23]. However, long-term HAART exposure leads to IL-17A restoration [23].

This novel study focused on the role of HAART on Th17 (IL-17A) in a homogenous population of pregnant normotensive and preeclamptic women, by comparing the serum concentrations of these cytokines in both study groups. This study may contribute to a better understanding of Th17 expression in the duality of HIV-associated normotensive and PE HAART-naïve (acute) and HAART-experienced (chronic) women.

## 2. Materials and Methods

### 2.1. Study Population and Sample Collection

Institutional ethical and hospital regulatory permissions were obtained for the study (Biomedical Research Ethics Committee, University of KwaZulu-Natal, South Africa (BCA338/17). After written consent was obtained, preeclamptic (PE) and normotensive (N) HIV-positive and HIV-negative Black South African pregnant women were recruited at a public health care hospital in South Africa. Normotensive (*n* = 90, age range: 18 ± 41 years) and PE (*n* = 96, age range: 18 ± 44 years) patients were recruited. Preeclampsia was defined as new-onset blood pressure of ≥140/90 mmHg taken on two occasions 4 hours apart and at least 1+ proteinuria measured by a urinary dipstick. Normotensive pregnant participants were defined as those with a blood pressure of ≤120/80 mmHg and without evidence of proteinuria. The relevant data of all research participants were obtained from their maternity case records. HIV testing was done after counselling using a rapid point-of-care test kit initially, as is the standard of care in South Africa. Maternal weight was categorised as normal weight (BMI: 18-25 kg/m^2^), overweight (BMI: 25-30 kg/m^2^), and obese (BMI: >30 kg/m^2^+).

To maintain ethnographic and anthropometric consistency, all patients recruited were of African ancestry and residents in the same geographical location. All participants were nonsmokers and nonconsumers of alcohol or recreational drugs, and all HIV-infected participants were on highly active antiretroviral therapy (HAART: tenofovir, emtricitabine, and efavirenz) as per South African National HIV guidelines [11]. HAART is the current recommended treatment for HIV infection. The current recommended treatment for HIV infection in pregnant and nonpregnant women is HAART [24]. The use of HAART in pregnancy is important for the reduction of perinatal transmission by several mechanisms, including lowering maternal antepartum viral load and preexposure and postexposure prophylaxis of the infant [25]. Women with chronic medical conditions were excluded from the study.

For analysis, women were grouped according to pregnancy type, gestational age, and HIV status. Women with PE were grouped into early onset, that is, ≤33 weeks + 6 days (*n* = 50; EOPE), and late onset ≥34 weeks of gestation (*n* = 50; LOPE).

The EOPE and LOPE groups were, respectively, subdivided by HIV status and duration of ARV therapy into HIV negative (*n* = 30), HIV acute (*n* = 10), and HIV chronic (*n* = 10). Similarly, the normotensive group was subdivided according to HIV status and duration of ARV therapy into HIV negative (*n* = 90), HIV positive acute (*n* = 30), and HIV positive chronic (*n* = 30).

HIV positive acute was defined as women who were on HAART treatment for the first time during pregnancy, and HIV positive chronic were defined as women who were previously on HAART before pregnancy.

### 2.2. Quantification of Human Cytokine Interleukin 17A (IL-17A)

Maternal blood samples collected in sterile serum separation tubes (Clot Activator: BD VAC PL PLAIN 6ML PE: 368815: Becton Dickinson) were centrifuged at 3000 rpm for 10 min at 4°C, and the supernatant was used for the quantification of Th1 (IL-2, IL-6, IL-12, IL-17, IFN-*γ*, and TNF-*α*), Th2 (IL-4, IL-5, IL-10, and IL-13), and Th17 (IL-17A) using the multiplex enzyme-linked immunosorbent assay. A Bio-Plex Pro™ Human Cytokine Standard 27-Plex, Group I kit was used according to the manufacturer's instructions (Bio-Rad Laboratories Inc., USA). The standards were prepared in a 1 : 10 and 1 : 4 dilution series, whilst samples were prepared in a 1 : 4 dilution. This bead-based flow cytometric assay allowed for multiplex analyses. The immunoassay involved the incubation of the antigen samples, i.e., Th1/Th2/Th17 with captured antibody-coupled beads. Subsequently, biotinylated detection antibodies coupled with a reporter conjugate, streptavidin-phycoerythrin (SA-PE), completed the interaction. The sample concentration was read using the Bio-Plex® MAGPIX™ Multiplex Reader (Bio-Rad Laboratories Inc., USA). Bio-Plex Manager™ software version 4.1 was used to analyse the data.

## 3. Statistical Analysis

GraphPad Prism 5.00 for Windows (GraphPad Software, San Diego, California, USA) was used to analyse the data. When the distribution was parametric, descriptive statistics for continuous data is presented by median, interquartile range (IQR), and mean ± standard deviation, whilst nonparametrically distributed data are presented as median and IQR. To determine statistical significance across all groups, a Mann–Whitney *U* or Kruskal-Wallis test in combination with Dunn's multiple comparison *post hoc* test was carried out. Statistical significance was *P* < 0.05.

## 4. Results

### 4.1. Clinical Characteristics of Participants

Table 1 provides a summary of the clinical and demographic features of the study population. As expected, systolic and diastolic blood pressures (BP) differed between the normotensive and PE groups (*P* ≤ 0.0001). Similarly, gestational age was statistically different between the normotensive pregnant and PE groups (*P* < 0.001 each; two-sample Wilcoxon rank-sum (Mann–Whitney) test). There were no significant differences in maternal weight (*P* = 0.1316), maternal height (*P* = 0.6761), BMI (*P* = 0.0638), and maternal age (*P* = 0.9574) between normotensive versus EOPE versus LOPE groups.

### 4.2. Serum Concentration Levels of IL-17A

These analytes were below the detection limits: Th1 (IL-2, IL-6, IL-12, IFN-*γ*, and TNF-*α*) and Th2 (IL-4, IL-5, IL-10, and IL-13).

#### 4.2.1. Pregnancy Type

**Figure 1:**
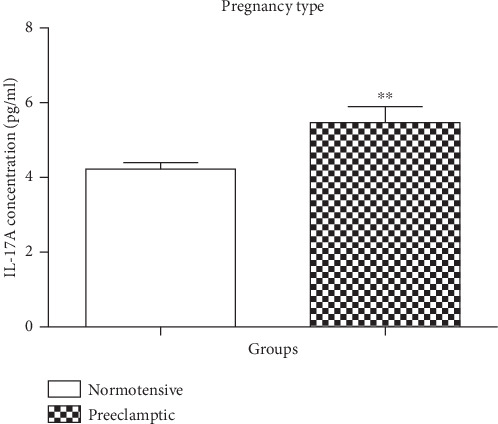
Serum concentration levels of IL-17 (pg/ml) according to pregnancy type. Normotensive (N) *vs.* preeclamptic (PE). Results are represented as median and interquartile range. ^∗∗^Serum concentration levels are significantly different between N and PE, *P* = 0.0014.

The concentration of IL-17A was statistically different between the N and PE groups (Mann–Whitney *U* = 881.5; *P* = 0.0014) Figure 1. The concentration of IL-17A was decreased in the N (mean = 4.228 pg/ml; 95% CI: 4.576–3.881) compared to the PE group (mean = 5.468 pg/ml; 95% CI: 6.332–4.604).

#### 4.2.2. Gestational Age

**Figure 2:**
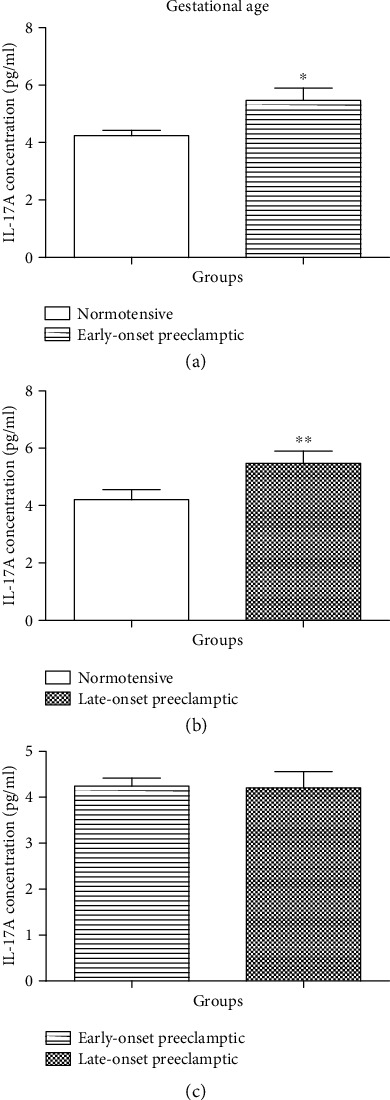
Serum concentration levels of IL-17 (pg/ml) according to gestational age. (a) Normotensive (N) vs. early-onset preeclamptic (EOPE), (b) N *vs.* LOPE, and (c) EOPE *vs.* LOPE. Results are represented as median and interquartile range. ^∗^Serum concentration levels are significantly different between N and EOPE, *P* = 0.0113. ^∗∗^Serum concentration levels are significantly different between N and LOPE, *P* = 0.0063. No significant difference in serum concentration levels between EOPE and LOPE.

The concentration of IL-17A was statistically different between N and EOPE (Mann–Whitney *U* = 567.5; *P* = 0.0113) Figure 2. There is a decrease in the concentration of IL-17A in the N group (mean = 4.243 pg/ml; 95% CI: 4.605–3.881) compared to the EOPE group (mean = 5.468 pg/ml; 95% CI: 6.332–4.604). Additionally, there was a significant difference in the levels of IL-17A between N and LOPE groups (Mann–Whitney *U* = 314.0; *P* = 0.0063) Figure 2. A decrease in the levels of IL-17A in N (mean = 4.205 pg/ml; 95% CI: 4.943–3.466) compared to the LOPE (mean = 5.468 pg/ml; 95% CI: 6.332–4.604) was observed. Furthermore, there was no significant difference in the levels of IL-17A between EOPE and LOPE (Mann–Whitney *U* = 333.0; *P* = 0.3341) Figure 2.

### 4.3. HIV Status

#### 4.3.1. HIV Negative

**Figure 3:**
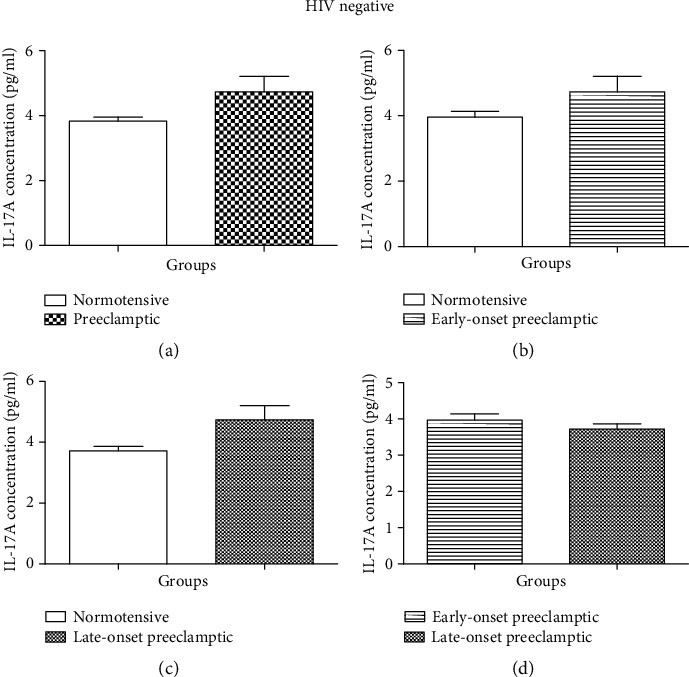
Serum concentration levels of IL-17 (pg/ml) according to HIV status. *HIV-negative groups*: (a) HIV-negative normotensive (N-) *vs.* HIV-negative preeclamptic (PE-); (b) N- *vs.* EOPE-; (c) N- *vs.* LOPE-; (d) EOPE- *vs.* LOPE-. Results are represented as median and interquartile range. No significant difference in serum concentration levels between N- *vs.* PE-, N- *vs.* EOPE-, and EOPE- *vs.* LOPE-. ^∗^Serum concentration levels are significantly different between N- and LOPE-, *P* = 0.0429.

No statistically significant effect was observed in IL-17A concentration between N and PE (Mann–Whitney *U* = 194.5; *P* = 0.1014) Figure 3. There is no statistical difference observed in IL-17 concentration between the N and EOPE groups (Mann–Whitney *U* = 103.5; *P* = 0.3266) Figure 3. A downward trend in IL-17A concentration was observed in the N group (mean = 3.967 pg/ml, 95% CI: 4.336–3.597) compared to the EOPE group (mean = 4.735 pg/ml; 95% CI: 5.748–3.723).

There is no statistical difference between the N and LOPE groups (Mann–Whitney *U* = 91.00; *P* = 0.0858) Figure 3. Moreover, the concentration of IL-17A was decreased in the N (mean = 3.719 pg/ml; 95% CI: 4.026–3.412) compared to the LOPE group (mean = 4.735 pg/ml; 95% CI: 5.748–3.723). There was no statistical significance in the concentration of IL-17A between EOPE and LOPE (Mann–Whitney *U* = 98.00; *P* = 0.3167) Figure 3.

#### 4.3.2. HIV Positive

**Figure 4:**
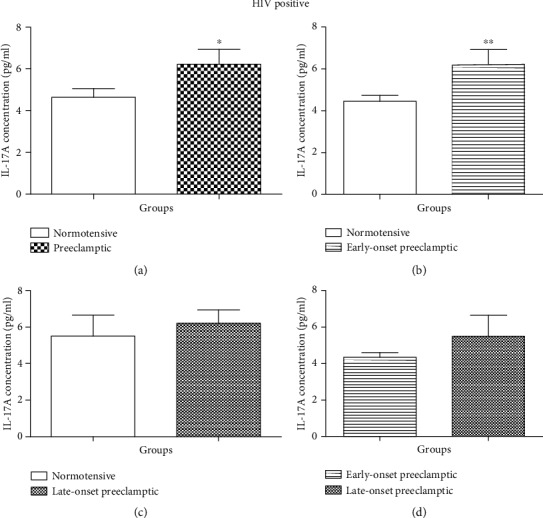
Serum concentration levels of IL-17 (pg/ml) according to HIV status. *HIV-positive groups*: (a) HIV-positive normotensive (N+) *vs.* HIV-positive preeclamptic (PE+); (b) N+ *vs.* EOPE+; (c) N+ *vs.* LOPE+; (d) EOPE+ *vs.* LOPE+. Results are represented as median and interquartile range. ^∗^Serum concentration levels are significantly different between N+ and PE+, *P* = 0.0114. ^∗∗^Serum concentration levels are significantly different between N+ and EOPE+, *P* = 0.0071. No significant difference in serum concentration levels between N+ *vs.* LOPE+ and EOPE+ *vs.* LOPE+.

A significant effect was observed in IL-17A concentration between HIV positive N and PE (Mann–Whitney *U* = 127.5; *P* = 0.0114) Figure 4. A downward trend in IL-17A concentration was observed in the N group (mean = 4.639 pg/ml, 95% CI: 5.516–3.762).

A significant difference was also noted in N *vs.* EOPE (Mann–Whitney *U* = 137.5; *P* = 0, 0071) Figure 4. A decrease in IL-17A concentration was noted in N (mean = 4.450 pg/ml, 95% CI: 5,033–3,867) compared to EOPE (mean = 6.220 pg/ml, 95% CI: 7679–4.761). Additionally, there was no statistically significant difference between N and LOPE (Mann–Whitney *U* = 88.00; *P* = 0.6724) Figure 4.

There was no statistical significance in the concentration of IL-17A between EOPE and LOPE (Mann–Whitney *U* = 48.00; *P* = 0.4355) Figure 4.

### 4.4. Duration of HAART

#### 4.4.1. HAART-Acute

**Figure 5:**
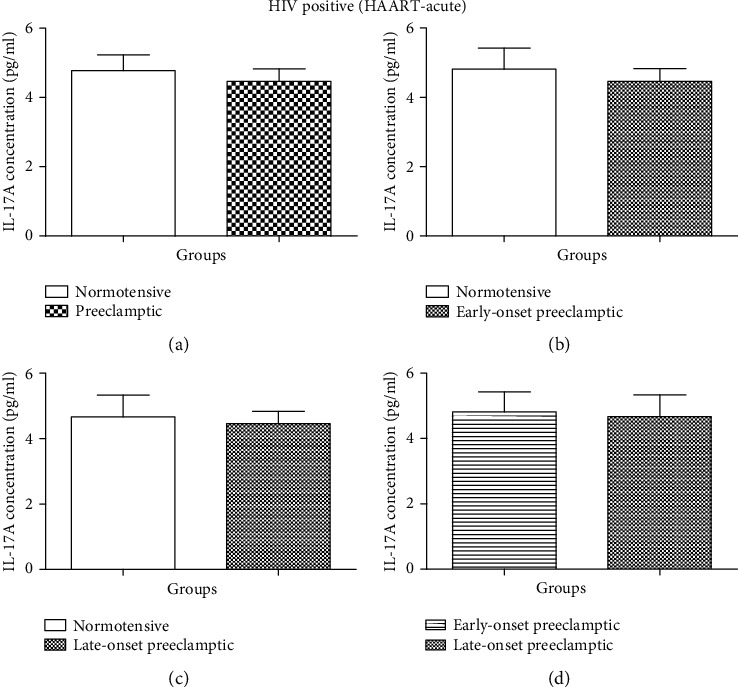
Serum concentration levels of IL-17 (pg/ml) according to HIV status. *HAART acute* groups. (a) Normotensive (N-acute) *vs.* preeclamptic (PE-acute); (b) N-acute *vs.* EOPE-acute; (c) N-acute *vs.* LOPE-acute; (d) EOPE-acute *vs.* LOPE-acute. Results are represented as median and interquartile range. No significant difference in serum concentration levels between all pregnancy types (N-acute *vs.* PE-acute, N-acute *vs.* EOPE-acute, N-acute *vs.* LOPE-acute, and EOPE-acute *vs.* LOPE-acute).

There was no statistical difference in IL-17A between N and PE groups (Mann–Whitney *U* = 69.5; *P* = 0.9256) Figure 5. Similarly, IL-17A concentration did not differ between N and EOPE women (Mann–Whitney *U* = 47.00; *P* = 0.3662) Figure 5. Nonetheless, a downward trend in IL-17A concentration was observed in N (mean = 4.462 pg/ml, 95% CI: 5.265–3.658) compared to EOPE (mean = 4.813 pg/ml; 95% CI: 6.259–3.366) groups. Likewise, there was no statistical difference observed in IL-17A concentration between N and LOPE (Mann–Whitney *U* = 17.00; *P* = 0.3886) Figure 5. Despite the nonsignificance, a downward trend in IL-17 concentration was observed between N (mean = 4.462 pg/ml, 95% CI: 5.265–3.658) and LOPE (mean = 4.667 pg/ml; 95% CI: 7.535–1.798) groups. No statistically significant effect was observed in IL-17A concentration between HIV acute EOPE and LOPE (Mann–Whitney *U* = 11.50; *P* = 0.5000) Figure 5.

#### 4.4.2. HAART-Chronic

**Figure 6:**
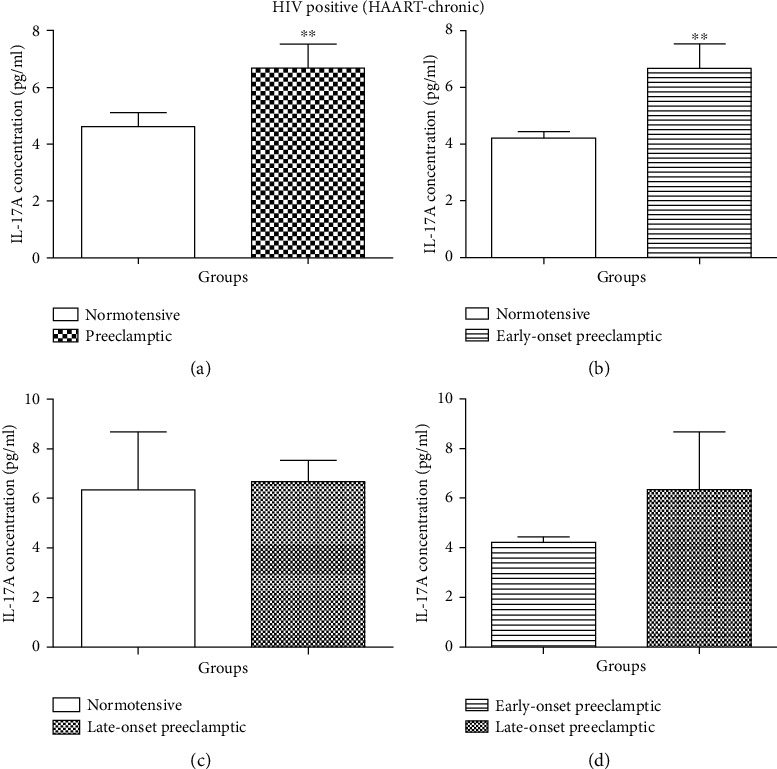
Serum concentration levels of IL-17 (pg/ml) according to HIV status: *HAART-chronic groups*. (a) Normotensive (N-chronic) *vs.* preeclamptic (PE-chronic); (b) N-chronic *vs.* EOPE-chronic; (c) N-chronic *vs.* LOPE-chronic; (d) EOPE-chronic *vs.* LOPE-chronic. Results are represented as median and interquartile range. ^∗∗^Serum concentration levels are significantly different between N-chronic vs. PE-chronic, *P* = 0.0062, and N-chronic *vs.* EOPE-chronic, *P* = 0.0029. No significant difference in serum concentration levels between N-chronic *vs.* LOPE-chronic and EOPE-chronic *vs.* LOPE-chronic.

The concentration of IL-17A was statistically different between the pregnancy types (N vs. PE groups) (Mann–Whitney *U* = 71.00; *P* = 0.0062) Figure 6. There was a decrease in the concentration of IL-17A in the N group (mean = 4.633 pg/ml; 95% CI: 5.682–3.584) compared to the PE (mean = 6.675 pg/ml; 95% CI: 8.466–4.884). There was also a statistical difference between N and EOPE (Mann–Whitney *U* = 46.00; *P* = 0.0029) Figure 6. There was a decrease in the concentration of IL-17A in the N group (mean = 4.208 pg/ml; 95% CI: 4.705–3.711) compared to the EOPE (mean = 6.675 pg/ml; 95% CI: 8.466–4.884). However, there was no statistical significance in the concentration of IL-17A between the N and LOPE (Mann–Whitney *U* = 24.00; *P* = 0.6915) groups.

There was no statistical significance in the concentration of IL-17A between EOPE and LOPE (Mann–Whitney *U* = 13.00; *P* = 0.4633) Figure 6.

## 5. Discussion

Interleukin 17 (IL-17), also known as (IL-17A), is a major, strongly proinflammatory cytokine produced by Th17 helper cells [26]. This cytokine has potent proinflammatory properties, which have been associated with the development of inflammatory processes, acute immunological graft rejection, and autoimmune diseases such as PE and HIV/AIDS.

### 5.1. Based on Pregnancy Type

In our study, we have evaluated the serum concentration levels of IL-17A based on pregnancy type (N vs. PE), gestational age (N vs. EOPE and N vs. LOPE), HIV status (negative vs. positive), and duration of HAART (acute vs. chronic) in Black South African women.

We demonstrated a significant increase in serum levels of IL-17A in preeclamptics compared to normotensives (*P* < 0.0014) Figure 1. Our study is in accordance with those reporting increased levels of IL-17A in pregnancies complicated by miscarriage, preterm birth, and PE compared to normotensive pregnancies [21, 27–31] implicating the role of this cytokine in the pathophysiology of PE. Interestingly, IL-17A has also been incriminated in causing placental oxidative stress then serves as a stimulus to modulate the renin angiotensin system (particularly AT1-AAs) thus leading to PE development [21]. Similarly, Yang et al. reported increased expression levels of IL-17A in PE placentas and serum compared to normotensives [32]. Additionally, Molvarec et al. also found increased circulating interleukin 17 levels in preeclampsia compared to healthy nonpregnant and pregnant normotensive women [29]. More interestingly, a study conducted by Barnie et al. in Chinese women showed increased plasma levels of IL-17A in preeclamptics compared to normotensives; they concluded that increased levels of IL7A in PE women may be produced by innate lymphoid cells 3 (ILC3). Innate lymphoid cells are a recently identified lymphocyte population (gastrointestinal tract cells known to produce cytokines such as IL-17A and/or IL-22) rather than Th17 cells, since they noted no significant difference in Th17 cells in PE women compared to normotensive women [33, 34]. Similarly, another study done by Cao et al. in the Chinese population found increased serum levels of IL-17A in PE compared to normotensive women; however, they associated their finding with Th17 cell activity [35]. Although the majority of these studies have been conducted in Chinese population, more studies are needed in the Black South African population in order to validate our findings.

Data on the levels of IL-17A based on pregnancy type (normotensive vs. PE) are discrepant; IL-17 has been suggested to play a role in maintaining pregnancy by inducing secretion of progesterone and increasing invasiveness of placental cells [36, 37]. Moreover, IL-17 receptor has been reported to be expressed on extravillous trophoblasts to allow successful invasion of spiral arteries [36]. Surprisingly, some studies have reported no significant difference in the levels of IL-17A between normotensive and preeclamptic pregnancies [38, 39]. Therefore, more studies are still needed in order to understand how this cytokine is regulated based on the pregnancy type.

### 5.2. Based on Gestational Age

We observed a significant increase in serum levels of IL-17A in EOPE compared to N (*P* < 0.013) Figure 2. The serum levels of IL-17A were also significantly higher in LOPE compared to N (*P* < 0.0063) Figure 2. We observed no significant difference in serum levels between EOPE and LOPE. Our findings suggested that decreased levels of IL-17A in normotensives might be strongly associated with the development of LOPE since we noticed a higher significant increase in LOPE compared to normotensives. Yang et al. reported that IL-17 have a positive association with the formation of proteinuria during late-onset PE; therefore, we speculated that this might be the reason why we observed a higher significance in LOPE when compared to normotensives [32].

### 5.3. Based in HIV Status

Th17 cells have been implicated in host defence against a variety of pathogens and are involved in the pathogenesis of autoimmune diseases. Recently, Th17 cells were shown to be reduced during HIV infection in humans and SIV infection in nonhuman primates [40].

Based on HIV status, we observed no significant difference in serum levels of IL-17A between HIV negative N *vs.* PE, N *vs.* EOPE, N *vs.* LOPE, and EOPE *vs.* LOPE Figure 3. However, we observed a significant difference in the levels of IL-17A in HIV-positive N *vs.* PE (*P* < 0.0114) and significant difference between N and EOPE (*P* < 0.0071) Figure 4. The levels of IL-17A were higher in PE compared to normotensives and significantly higher in EOPE compared to normotensives. These findings suggest that in the presence of HIV infection, IL-17A is involved in the pathophysiology of EOPE; however, the mechanism is unknown. To the best of our knowledge, no studies have reported on IL-17A based on gestational age. Normally, both pregnancy and HIV infection are marked by an increase in anti-inflammatory response. However, upon HAART administration, proinflammatory cytokine response is activated [13]. IL-17A is a proinflammatory cytokine, and proinflammatory response is known to induce the development of PE [11]. Studies have reported that administration of HAART in pregnant women predisposes them to PE development [41, 42]. Therefore, we speculated that increased levels on IL-17A in EOPE were due to chronic HAART exposure. We therefore evaluated IL-17A based on the duration of HAART.

### 5.4. Based on HAART Duration

Studies have reported that HIV-infected women have higher risk of PE development than HIV-naïve women [14, 43]. Both HIV and PE are immune conditions, and several inflammatory cytokines play a role in the progression of these disorders. It is known that during the progression of HIV infection, Th2 response is activated; however, this is counteracted by the use of HAART. During the pathogenesis of PE, there is an increase in proinflammatory cytokines.

Several studies have documented that there is a decline in the levels of IL-17A in HIV-infected individuals [23, 44, 45]; however, upon HAART exposure, the levels of IL-17A become restored. Based on the duration of HAART, we found no significant difference in the serum levels of IL-17A between pregnancy type and gestational age in HAART-acute women Figure 5. This is due to the fact that during the early stages of HIV infection, the levels of IL-17A in HAART-naïve women are almost the same to those of HIV-negative individuals.

Interestingly, we found a significant increase in the serum levels of IL-17A in HAART-chronic women between N and PE (*P* < 0.0062) and between N and EOPE (*P* < 0.0029) Figure 6. The levels of IL-17A in PE were higher in PE compared to normotensives and in EOPE compared to normotensives. However, we noticed no significant difference between N and LOPE and between EOPE and LOPE. During the first trimester (placentation), Th17 cells play a role in promoting proliferation of trophoblast cells by secreting IL-17A [22]. Therefore, it is possible that chronic HAART exposure contributes to exaggerative secretion of IL-17A at this stage thus leading to the development of EOPE.

### 5.5. Study Limitations

CD4 counts were not available for all women as it is not a standard of practice in the public health institutions in SA; hence, the levels of IL-17A in our study could not be correlated with severity of HIV infection. Our findings are preliminary and limited by a small sample size. A larger cohort will enable further research. Further, the role of IL-17A in normotensive versus preeclamptic pregnancies needs further investigation, and peripheral expression of IL-17A needs to be collated with measures in the placenta. This study was restricted to SA Black women; thus, our findings may not necessarily apply to other ethnic groups.

## 6. Conclusion

Our findings suggested that IL-17A play a role in the pathophysiology of preeclampsia and that in the presence of HIV infection, chronic HAART exposure is associated with increased levels of IL-17A which might be associated with the pathophysiology of EOPE. The level of IL-17A can be used as a predictive risk indicator in HIV-associated PE.

## 7. Strengths

Previous studies have mainly focused on the role of IL-17A based on pregnancy type (N vs. PE) or HIV status (positive vs. negative). To our knowledge, this is the first study to report the expression of IL-17A in Black South African women based on pregnancy type, gestational age, HIV status, and duration of HAART.

## 8. Future Studies

More studies should be done on the role of IL-17A based on pregnancy type, gestational age, HIV status, and duration of HAART in different ethnic groups in order to see how this cytokine is regulated in other ethnic groups. Additionally, since serum levels of IL-17A cytokines were tested from peripheral circulating blood rather than fetomaternal interface, future studies should be done on serum from the fetomaternal interface since the primary source of the pathogenesis of preeclampsia arises from the placenta.

## Figures and Tables

**Table 1:** Patient demographic features of the study groups (normotensive = 150, early‐onset preeclampsia = 50, and late‐onset preeclampsia = 50).

Variables	Groups	Median	Q1-Q3	Mean ± SD	*P* value
Maternal weight (kg)	N	77	65-100	81.92 ± 18.35	
	EOPE	79	67.50-100.5	86.85 ± 30.31	0.1316
	LOPE	96.50	72.50-113.0	93.15 ± 21.95	

Maternal height (m)	N	157	153.25-163	157.5 ± 7.242	
	EOPE	159	154.5-164	158.8 ± 7.967	0.6761
	LOPE	160	155-164	159.1 ± 6.934	

BMI (kg/m^2^)	N	32.05	25.72-38.65	32.59 ± 7.301	
	EOPE	31.64	25.80-39.78	33.52 ± 9.483	0.0638
	LOPE	38	32.93-41.50	37.20 ± 8.021	

Systolic blood pressure (mmHg)	N	109	98.25-113.75	108.0 ± 11.25	
	EOPE	146	144-157	149.9 ± 10.17	^∗∗∗^<0.0001
	LOPE	145	140-149.75	145.40 ± 7.35	

Diastolic blood pressure (mmHg)	N	65.5	61-72	65.52 ± 9.38	
	EOPE	95	90-104	96.70 ± 9.20	^∗∗∗^<0.0001
	LOPE	94	90-98	93.25 ± 5.87	

Gestational age (weeks)	N	35	26-38	31.88 ± 6.73	^∗∗∗^<0.0001
	EOPE	24	20-30	24.25 ± 5.77	
	LOPE	36	35-37.25	35.95 ± 1.96	

Maternal age (years)	N	28	25-32.75	28.60 ± 5.90	
	EOPE	28.5	22.75-34.25	28.19 ± 7.35	0.9574
	LOPE	29	24-32.5	28.45 ± 7.13	

N: normotensive; EOPE: early-onset preeclampsia; LOPE: late-onset preeclampsia.

## Data Availability

The datasets generated during and/or analysed during the current study are available from the corresponding author on reasonable request.

## Abbreviations

BMI: Body mass index
EOPE: Early-onset preeclamptic
EOPE-: HIV-negative early-onset preeclamptic
EOPE+: HIV-positive early-onset preeclamptic
EOPE-acute: Early-onset preeclamptic HAART-acute
EOPE-chronic: Early-onset preeclamptic HAART-chronic
HAART: Highly active antiretroviral therapy
HELLP syndrome: Haemolysis, elevated liver enzymes, low platelet count syndrome
HIV: Human immune deficiency virus
IFN-*γ*: Interferon gamma
IL-1*β*: Interleukin 1-beta
IL-2: Interleukin 2
IL-6: Interleukin 6
IL-8: Interleukin 8
IL-12: Interleukin 12
IL-17A: Interleukin 17A
LOPE: Late-onset preeclamptic
LOPE-: HIV-negative late-onset preeclamptic
LOPE+: HIV-positive late-onset preeclamptic
LOPE-acute: HAART-acute late-onset preeclamptic
LOPE-chronic: HIV-positive (HAART-chronic) late-onset preeclamptic
N: Normotensive
N-: HIV-negative normotensive
N-acute: HAART-acute normotensive
N-chronic: HAART-chronic normotensive
NHLS: National Health Laboratory Services
PE: Preeclampsia
PE-: HIV-negative preeclamptic
Th1: Proinflammatory cytokines
Th2: Anti-inflammatory cytokines
TNF-*α*: Tumor necrotic alpha.

## Additional Points

*Clinical Significance*. Regardless of HIV status, IL-17A is elevated in normotensives compared to women with PE, highlighting its role in normal pregnancy maintenance. The upregulation of IL-17A in HIV-positive PE women may be attributed to prolonged HAART exposure, rather than to PE aetiology.
